# Spontaneous Intercostal Artery Bleeding in a Patient With Buerger's Disease: A Case Report

**DOI:** 10.7759/cureus.60447

**Published:** 2024-05-16

**Authors:** Faraz Badar, Harith Al-Ataby, Mohammed Al-Azzawi, Mohamed Omballi

**Affiliations:** 1 Pulmonary and Critical Care Medicine, The University of Toledo, Toledo, USA

**Keywords:** buerger disease, thoracotomy, transcatheter arterial embolization, retropleural hematoma, hemothorax, spontaneous intercostal artery bleeding

## Abstract

Intercostal artery (ICA) injury and bleeding are well-known complications of thoracic procedures and trauma; however, spontaneous ICA bleeding is a rare condition usually associated with specific underlying disorders that typically lead to the weakening of vasculature. Herein, we present a 42-year-old male with a history of Buerger's disease who developed spontaneous bleeding of the second left ICA after undergoing lower limb angioplasty. The bleeding was complicated by a large hemothorax and retropleural hematoma, resulting in hemorrhagic shock that necessitated massive transfusion, embolization, and eventual thoracotomy with evacuation.

## Introduction

Intercostal artery (ICA) bleeding is an uncommon yet potentially fatal condition, which can cause hemorrhagic shock and respiratory failure [1]. It is commonly associated with thoracic trauma and iatrogenic injuries. Spontaneous ICA bleeding is extremely rare and is typically reported only in patients with certain underlying disorders, such as connective tissue diseases, liver cirrhosis, coarctation of the aorta, or uncontrolled hypertension [2-6]. Only a limited number of cases of spontaneous ICA bleeding in the absence of these underlying disorders have been reported in the literature [1]. 

In this case report, we present a patient with Buerger's disease who developed spontaneous bleeding of the second left ICA after undergoing lower limb angioplasty. The patient was successfully treated with transcatheter arterial coil embolization (TAE) followed by thoracotomy and hematoma evacuation. 

## Case presentation

**Figure 1:**
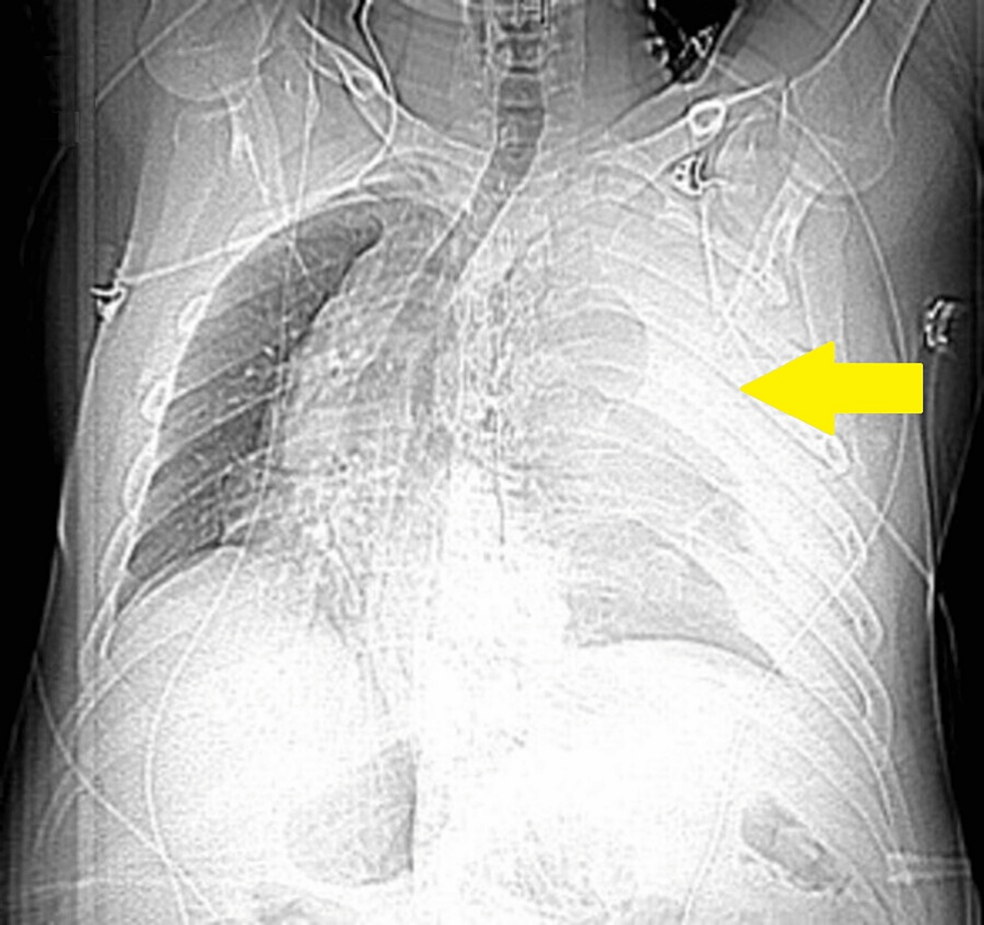
Chest X-ray demonstrating complete opacification of the left hemithorax with a right mediastinal shift.

**Figure 2:**
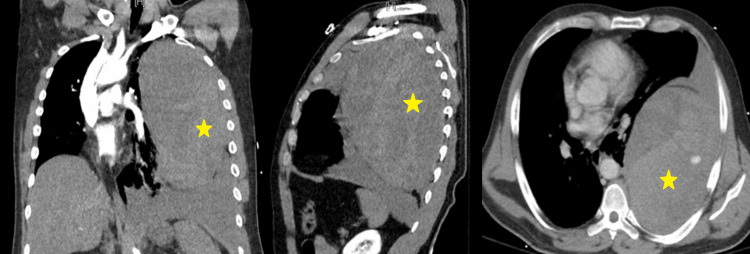
CT imaging demonstrating a large left hemothorax and retropleural hematoma, accompanied by right mediastinal shift, as observed in the coronal, sagittal, and axial planes, respectively.

A 42-year-old man with a history of Buerger's disease and polysubstance abuse presented to our hospital with critical limb-threatening ischemia involving the right lower leg. The patient underwent a right lower limb angiogram and recanalization of the occluded distal peroneal artery with balloon angioplasty under conscious sedation. Intraoperatively, he received 11,000 units of intravenous heparin. Postoperatively, in the recovery unit, as sedation wore off, the patient became severely agitated and was attempting to jump out of his bed. However, he then suddenly became unresponsive and went into shock, with a blood pressure of 70/30 mmHg and a heart rate of 130 beats/minute. He was emergently intubated, and resuscitation was initiated with intravenous fluids and vasopressors. Auscultation of the chest revealed absent breath sounds on the left side. Laboratory investigations were significant for a drop in the hemoglobin level from 11 g/dL preoperatively to 7 g/dL postoperatively and an increase in the serum lactate level to 5 mmol/L (Table 1). Chest X-ray showed complete opacification of the left hemithorax (Figure 1). Computed tomography angiography (CTA) of the chest showed a large left hemothorax and a left retropleural hematoma with linear contrast extending from the second left posterior ICA, compatible with acute hemorrhage (Figure 2). A mediastinal shift was also noted.

**Table 1:** Laboratory values. WBC, white blood cells; INR,  International Normalized Ratio; PT, prothrombin time; PTT, partial thromboplastin time; BUN, blood urea nitrogen

Hematology	Result	Reference range
WBC	13.1	3.5-10.8 K/µL
Hemoglobin	7.0	11.5-15.5 g/dL
Hematocrit	22.1	34.5%-45%
Platelet count	396	150-400K/µL
INR	1.2	0.8-1.2
PTT	27.8	25.1-35.7 s
General chemistry		
Sodium	136	136-145 mmol/L
Potassium	4.1	3.3-5.1 mmol/L
Chloride	105	98-107 mmol/L
Bicarbonate	28	22-29 mmol/L
BUN	18	8-23 mg/dL
Creatinine	1.3	0.7-1.2 mg/dL
Glucose	96	74-109 mg/dL
Magnesium	1.8	1.6-2.6 mg/dL
Phosphorus	3.5	2.5-4.5 mg/dL
Lactate	5.0	2-4 mmol/L

**Figure 3:**
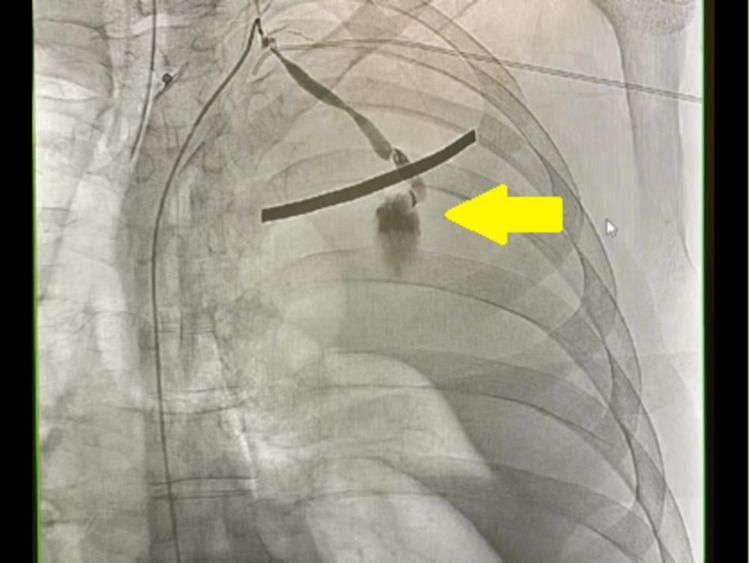
Angiogram of the left subclavian artery demonstrating the bleeding origin to be the left posterior second intercostal artery (ICA).

**Figure 4:**
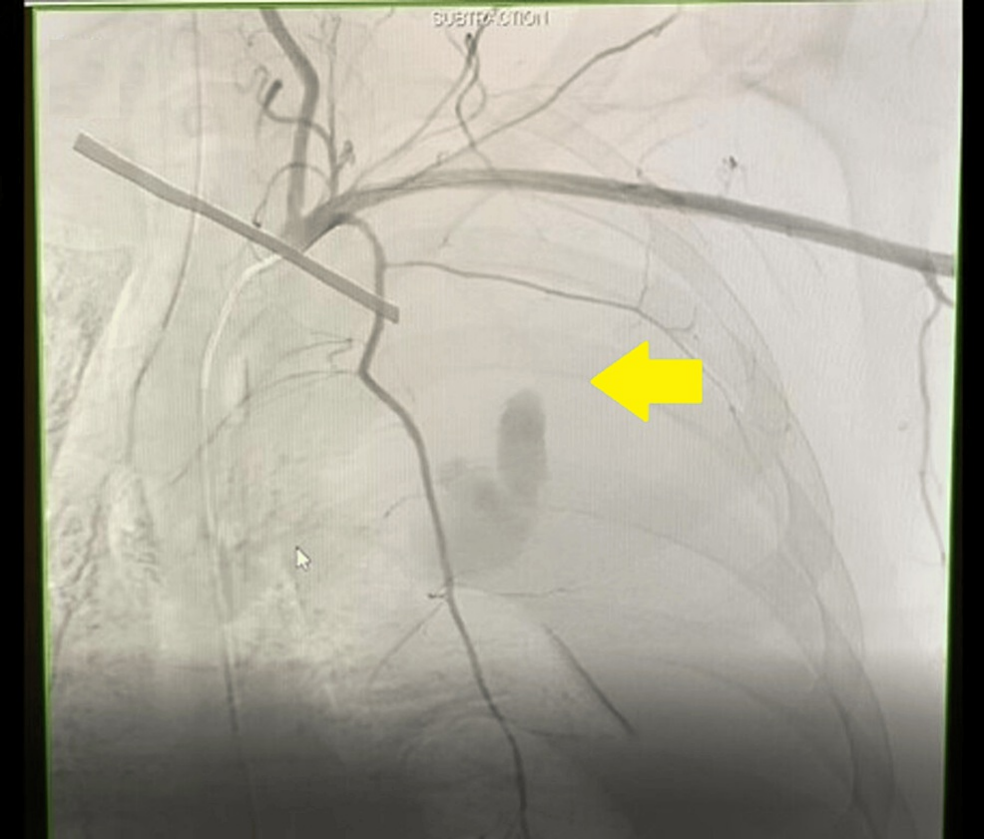
Successful hemostasis after transcatheter arterial coil embolization (TAE).

**Figure 5:**
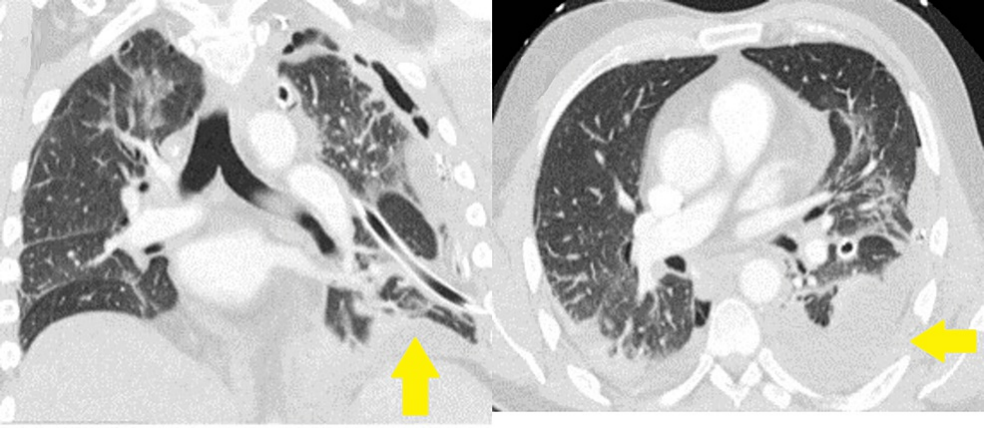
CT scan five days post thoracotomy showing interval improvement in the left-sided hemothorax and retropleural hematoma.

**Figure 6:**
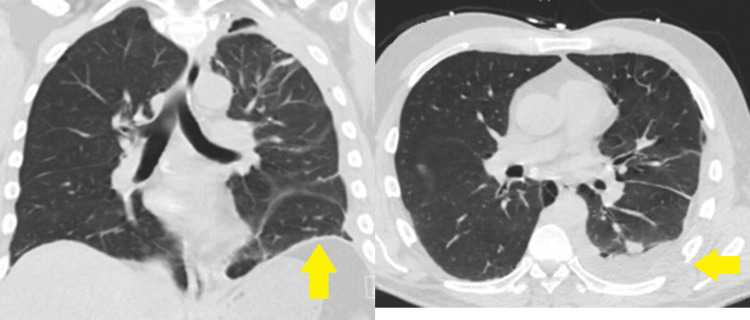
CT scan 10 days post thoracotomy showing near-complete resolution of the left-sided hemothorax and retropleural hematoma along with re-expanded left lung.

Massive transfusion protocol was initiated, and the patient received four units of packed red blood cells, one unit of platelets, and one unit of fresh frozen plasma. An angiogram of the left subclavian artery revealed the bleeding to be originating from the second left posterior intercostal artery (Figure 3). TAE was performed, and bleeding was successfully stopped (Figure 4). On the second day, thoracotomy and hematoma evacuation were performed. Two left-sided surgical chest tubes were placed, one in the retroperitoneum, while the other in the pleural space. A total of 2.5 L of frank blood was drained. The following day, the patient’s hemodynamics improved, and he was successfully extubated to room air. At this point, minimal fluid was draining from the chest tubes, and thus, both were removed. The patient was discharged and advised to follow up in pulmonary and vascular surgery outpatient clinics. CT scans of the chest were repeated five and 10 days after thoracotomy, and they showed significant improvement with expansion of the left lung (Figure 5), and resolution of the left-sided hemothorax and retropleural hematoma (Figure 6).

## Discussion

Penetrating or blunt thoracic trauma is the most common cause of ICA injury [7,8]. Trauma can result in rib fractures, which can damage the adjacent structures, such as intercostal and internal mammary arteries [9]. ICA rupture can sometimes be iatrogenic in nature and may occur during elective and emergency thoracic procedures, including thoracoscopy, thoracocentesis, pleural biopsy, and chest drain insertion [7]. 

However, spontaneous bleeding from the ICA is extremely rare. Izumoto et. al reported less than 10 cases in the literature [1]. It is usually associated with conditions that cause the arterial walls to become weak. Some of these conditions include neurofibromatosis type 1 (NF1), Ehlers-Danlos syndrome, Marfan syndrome, systemic lupus erythematosus, cirrhosis, and uncontrolled hypertension [2-6]. NF1 is thought to affect nerve tissue as well as systemic vasculature and is the most common cause of spontaneous ICA bleeding [2,8,9]. However, the precise mechanism behind ICA rupture in these patients is not understood. Episodes of violent cough, use of anti-platelets and anticoagulants, and pulmonary infections may also predispose to the development of spontaneous ICA bleeding [6,10,11]. In addition, older individuals may be at an increased risk of spontaneous rupture due to an increase in the tortuosity of intercostal arteries and traversing collaterals with age [7].

Presenting symptoms for spontaneous ICA bleeding are variable and include chest pain, back pain, flank pain, neck pain, and dyspnea. About half of the reported patients developed hemodynamic instability and later went into shock [1]. The 10th and 11th ICAs have been reported as the most common location of spontaneous rupture [8]. However, in our patient, the bleeding was found to originate from the second left posterior ICA.

The diagnosis of ICA rupture is usually based on the presence of a massive hemothorax or chest wall and/or retroperitoneal hematoma. Pleural fluid can be confirmed by performing chest radiography; however, CT scanning is an important diagnostic tool and is reported to be the preferred imaging modality in reported cases [1,12]. Contrast-enhanced CT and CTA are also useful, as they can detect the bleeding vessel involved as well as the associated complications. They can also rule out potential causes, such as rib fractures. A decrease in the hemoglobin level, as was seen in our patient, should also alert physicians to the possibility of this diagnosis [6,11].

Spontaneous rupture of an ICA can be fatal secondary to hemorrhagic shock, and therefore, early recognition and treatment are critical. Angiography and therapeutic embolization are effective [1,6]. TAE is a minimally invasive procedure that can be performed immediately and is commonly used for the emergency management of bleeding caused by traumatic ICA injuries [13,14]. It has also been reported as the preferred first-line treatment for spontaneous ICA bleeding [1]. If TAE cannot be performed or fails to control bleeding, then surgical exploration is an option [1,15]. Strategies for surgical exploration vary based on the clinical scenario and hemodynamic status of the patient. For instance, thoracotomy followed by placement of hemostatic clips, suture ligation, and drain placement have all been reported [7,15].

Our patient was young and lacked any history of exposure to external stimuli, such as trauma or chest surgery. Moreover, he did not have any underlying connective tissue disease that causes the weakening of arterial walls and subsequently leads to spontaneous ICA bleeding. It is important to note that our patient did have a history of Buerger's disease or thromboangiitis obliterans, which is an inflammatory disease that affects the small- and medium-sized arteries and veins [16]. However, vessels of the distal extremities are usually involved. In addition, the acute phase of Buerger's disease is typically characterized by the presence of an occlusive inflammatory thrombus with relative sparing of the vessel walls and internal elastic lamina [17]. Thus, this condition has not been associated with spontaneous ICA rupture and bleeding. Physical examination and imaging of our patient did not reveal any evidence of thoracic trauma. Based on these findings, our patient was diagnosed with idiopathic spontaneous ICA bleeding.

The exact etiology of ICA bleeding in our patient cannot be explained; however, we believe that anticoagulation with full-dose heparin for lower limb angioplasty may have contributed to the development of spontaneous bleeding in the absence of any underlying disorders. Moreover, when sedation subsided postoperatively, he became agitated, likely from withdrawal, given his history of polysubstance abuse. Extreme changes in the intrathoracic pressure secondary to violent, forceful body movements may have played a role in the rupture of the ICA. Our patient went into hemorrhagic shock but was successfully managed with TAE due to early recognition of the source of bleeding using CTA.

## Conclusions

Spontaneous ICA rupture and bleeding are rare and potentially fatal. It may be complicated by hemothorax and/or hematoma formation, which can result in respiratory failure and hemorrhagic shock. CT imaging plays a critical role in the diagnosis. Angiography and TAE are considered the first-line treatment for this condition. However, surgical exploration may be required if TAE fails or is unavailable. Our case report not only highlights the significance of early recognition and prompt treatment for this life-threatening condition but also appears to be the first reported case associated with Buerger's disease. 
